# Cholesterol Depletion Inactivates XMRV and Leads to Viral Envelope Protein Release from Virions: Evidence for Role of Cholesterol in XMRV Infection

**DOI:** 10.1371/journal.pone.0048013

**Published:** 2012-10-26

**Authors:** Yuyang Tang, Alvin George, Thyneice Taylor, James E. K. Hildreth

**Affiliations:** 1 Department of Molecular and Cellular Biology, College of Biological Sciences, University of California Davis, Davis, California, United States of America; 2 Department of Medicine, School of Medicine, Vanderbilt University, Nashville, Tennessee, United States of America; Institut Pasteur Korea, Republic of Korea

## Abstract

Membrane cholesterol plays an important role in replication of HIV-1 and other retroviruses. Here, we report that the gammaretrovirus XMRV requires cholesterol and lipid rafts for infection and replication. We demonstrate that treatment of XMRV with a low concentration (10 mM) of 2-hydroxypropyl-β-cyclodextrin (2OHpβCD) partially depleted virion-associated cholesterol resulting in complete inactivation of the virus. This effect could not be reversed by adding cholesterol back to treated virions. Further analysis revealed that following cholesterol depletion, virus-associated Env protein was significantly reduced while the virions remained intact and retained core proteins. Increasing concentrations of 2OHpβCD (≥20 mM) resulted in loss of the majority of virion-associated cholesterol, causing disruption of membrane integrity and loss of internal Gag proteins and viral RNA. Depletion of cholesterol from XMRV-infected cells significantly reduced virus release, suggesting that cholesterol and intact lipid rafts are required for the budding process of XMRV. These results suggest that unlike glycoproteins of other retroviruses, the association of XMRV glycoprotein with virions is highly dependent on cholesterol and lipid rafts.

## Introduction

Within the bilayer of the plasma membrane, cholesterol is enriched along with sphingolipids and glycerophospholipids in specialized microdomains known as lipids rafts. Several studies have demonstrated that lipid rafts play important roles in the replication of a number of enveloped viruses, including human immunodeficiency virus type 1 (HIV-1) [1], human T-cell leukemia virus type 1 (HTLV-1) and other enveloped viruses [2], [3], [4], [5], [6], [7]. Studies from our laboratory and others have suggested that lipid rafts are involved in both early and late phases of the HIV-1 replication cycle [1], [8], [9], [10], [11], [12], [13]. Depletion of membrane cholesterol with low concentrations of β-cyclodextrin is an effective, non-toxic method for dispersing lipid rafts [14]. We have reported that partial depletion of virion-associated cholesterol with 2-hydroxypropyl-β-cyclodextrin (2OHpβCD) limits membrane fusion and abrogates HIV infectivity. Further, extensive cholesterol depletion with 2OHpβCD causes dissociation of microdomain components from viral membranes. This results in non-infectious permeablized virions that have lost mature viral core proteins and lipid raft markers but retain envelope glycoproteins [8], [9]. Treating HIV-susceptible cells with 2OHpβCD also inhibits HIV-1 infection. Treating virus-producing cells with 2OHpβCD or other cholesterol-reducing agents to disrupt lipid rafts inhibits virus production and reduces the infectivity of the released virions [9], [12]. Thus cholesterol and lipid rafts are involved in both HIV-1 entry and assembly. Furthermore, cholesterol and lipid rafts are involved in HIV-1 replication at the level of both virus and target cell.

Murine leukemia viruses (MLV) are retroviruses belonging to the genus Gammaretrovirus that cause cancers and other diseases in mice. MLV shares a number of properties with HIV-1, and like HIV-1, the membrane of MLV is enriched in cholesterol and raft-specific sphingolipids [15]. In addition, the Env and core proteins of both ecotropic MLV (E-MLV) and amphotropic MLV (A-MLV) have been reported to associate with lipid rafts [16], [17], [18], suggesting that lipid rafts and cholesterol are involved in MLV assembly and/or release. It has been reported that like HIV-1, entry of MLV into susceptible cells is lipid raft-dependent. The receptors of E-MLV and A-MLV have been shown to be associated with caveolin, a lipid raft component, and disruption of cellular lipid rafts by cholesterol depletion reduced cell susceptibility to MLV infection [19], [20].

Xenotropic murine leukemia virus-related virus (XMRV) is a gammaretrovirus that shares 94% overall sequence homology with MLV. XMRV was originally detected in human prostate cancer tissues [21]. While some subsequent studies reported the presence of XMRV in prostate cancer and chronic fatigue syndrome samples, several other studies found little or no evidence of the virus in patient samples [22], [23], [24], [25]. Recent reports indicate that XMRV arose as a result of a recombination event between two endogenous MLV during xenotransplantation of cells in nude mice [26]. The available data now indicates that it is unlikely that XMRV is associated with human disease. Nonetheless studies designed to understand the role of cholesterol and lipid rafts in the biology of XMRV as a model gammaretrovirus are highly relevant given the important role of cholesterol and lipid rafts in the biology of other retroviruses. Insights gained studying XMRV as a model system may provide new approaches to treating retroviruses that are associated with disease. In addition, other gammaretroviruses may arise in the future with the potential to infect humans and cause disease.

In the present study, we examined the effect of 2OHpΒCD on the infectivity and membrane integrity of XMRV. Our study reveals that depletion of cholesterol from XMRV particles resulted in loss of Env protein and abrogated virus infectivity. These results indicate that virus-associated cholesterol is necessary to maintain the XMRV membrane structure required for infectivity. In addition, the release of XMRV from LNCaP cells was inhibited by 2OHpΒCD treatment, suggesting that XMRV budding and/or release occurs at lipid rafts as previously established for HIV-1.

## Results

### Removal of Cholesterol Completely Inactivates XMRV

**Figure 1:**
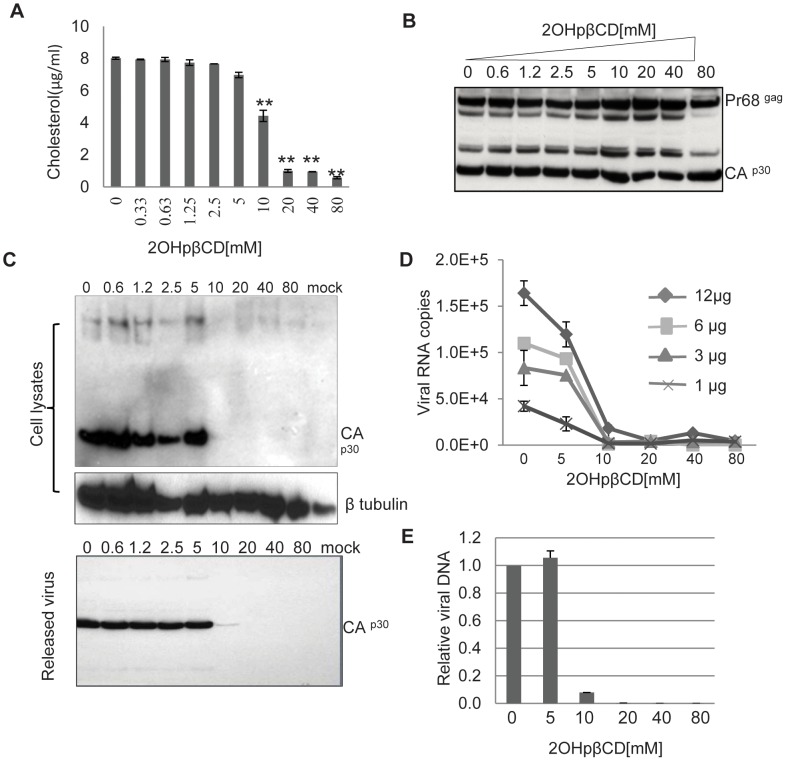
Depletion of cholesterol abolishes XMRV infectivity. Purified virus was incubated with indicated concentrations of 2OHpβCD in RPMI 1640 medium for 1 hr at 37°C and the treated virus was pelleted. (**A**) The residual cholesterol in 2OHpβCD-treated virions was measured by Amplex Red assay. Error bars reflect standard derivation of three independent experiments. **, *P*<0.001, compared to the virions without 2OHpβCD treatment. (**B**) The infectivity of 2OHpβCD-treated XMRV was tested in LNCaP cells. Comparable amounts of treated virus used to infect LNCaP cells were confirmed by immunoblot with an anti-MLV goat serum. (**C**) Cell lysates and virus-containing supernatants of infected LNCaP cells were harvested at 48 hrs of culture after exposing cells to virus and subjected to western blot analysis. Top panel: cell lysate from infected cells was analyzed with goat antiserum to MLV p30 Gag or β tubulin; bottom panel: released virus in the supernatant was analyzed with goat antiserum to MLV p30 Gag. Mock: cells exposed to medium alone without virus. Data are representative of five independent experiments (**D**) LNCaP cells were infected with 12, 6, 3 and 1 µg (total protein) of 2OHpβCD-treated XMRV as indicated. The released virus in the cell supernatants was harvested at 48 hrs post-infection and quantified by qRT-PCR. (**E**) LNCaP cells were infected with 12 µg (total protein) of 2OHpβCD-treated XMRV. After 48 hrs, proviral DNA from the infected cells was quantified by Q-PCR. Quantified data are normalized to cells infected untreated control virus (arbitrarily set as 1). Error bars reflect standard derivation of three independent experiments (D, E).

Virion associated cholesterol has been shown to be critical in retrovirus infection. We and others have reported that depletion of virion-associated cholesterol abrogates HIV infectivity ([8], [9], [12]. To investigate the role of virion-associated cholesterol and lipid rafts in XMRV replication, virus preparations were exposed to 2OHpβCD at concentrations ranging from 0.325 to 80 mM for 1 hr at 37°C, and the amount of cholesterol remaining in virions was determined with the Amplex® Red Cholesterol Assay (Figure 1A). Treatment of virions with up to 5 mM 2OHpβCD had little or no effect on virion-associated cholesterol. There was a 30–40% reduction in virion-associated cholesterol after treatment with 10 mM 2OHpβCD. Following treatment with 2OHpβCD at 20 mM or above, the level of virion-associated cholesterol further declined to 10–20% of the untreated control (Figure 1 A).

We then determined the infectivity of control and 2OHpβCD-treated virus preparations. The recovered virus samples were tested for infectivity using equal amounts of input virus, normalized to total protein (Figure 1B). LNCaP cells were exposed to virus preparations treated with medium or various concentrations of 2OHpβCD and the input virus was removed after 8 hrs by extensive washing. The cells were then cultured for 48 hrs before cell lysates and culture supernatants were analyzed by immunoblotting, qRT-PCR or qPCR. While the XMRV capsid protein (p30) was readily detected in cells infected with untreated control virus or with virus preparations treated with 2OHpβCD at concentrations between 0.625 mM and 5 mM, the p30 protein was not detected in cells infected with virus preparations treated with 2OHpβCD at concentrations of 10 mM or above (Figure 1C, top panel). Western blot analysis of the culture supernatants from infected cells indicates that very little or no virus was released from cells exposed to XMRV treated with 2OHβBCD at 10 mM or above (Figure 1C, bottom panel). qRT-PCR analysis showed that viral RNA was not detected or was present at extremely low levels in culture supernatants from cells infected with XMRV preparations treated with 2OHpβCD at concentrations of 10 mM or above (Figure 1D). Similar findings were obtained when proviral DNA was measured in infected cells (Figure 1E). These data indicate that depletion of cholesterol abolished the infectivity of XMRV virions.

**Figure 2:**
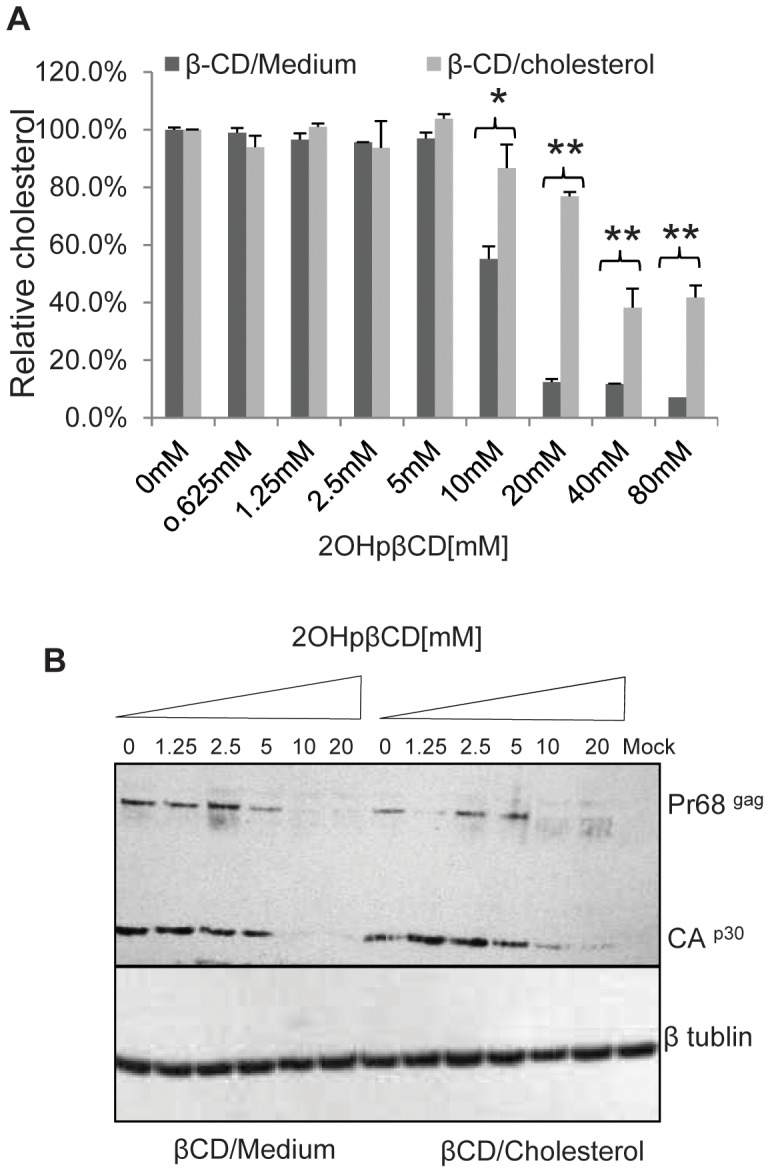
Exogenous cholesterol does not restore infectivity of 2OHpβCD-treated XMRV. After treating XMRV with 2OHpβCD, the virus was pelleted by ultracentrifugation. The virus was resuspended in RPMI alone (β-CD/medium) or RPMI containing cholesterol (48 µg/ml as a complex with 1 mM 2OHpβCD) (β-CD/Cholesterol) and incubated 1 hr at 37°C. (**A**) The cholesterol in virions was measured by Amplex Red assay. Data normalized to mock treated control (0 mM arbitrarily set as 100%). Error bars reflect standard derivation of three independent experiments. *, *P*<0.05; **, *P*<0.001, comparison between virions treated with β-CD/medium or treated with β-CD/Cholesterol. (**B**) Viral infectivity was then assayed on LNCaP cells by Western blot with goat antiserum against MLV p30 Gag as described before. Data are representative of three independent experiments. Mock: cells exposed to medium alone without virus.

Previous studies from our laboratory have demonstrated that the effect of 2OHβBCD on HIV-1 infectivity could be reversed by exposing treated virions to exogenous cholesterol thereby partially restoring the cholesterol content of the particles [9]. To determine if we could replicate this effect on XMRV, we treated XMRV with various concentrations of 2OHpβCD, recovered the virions by ultracentrifugation, then incubated the virions in RPMI medium alone or medium containing 48 µg/ml of cholesterol as a complex with 2OHpβCD (1 mM) for 1 hr at 37°C. After the virions were re-purified, cholesterol assays showed that this treatment restored cholesterol to 80–90% of control levels in viruses treated with 10 mM and 20 mM 2OHpβCD (Figure 2A). As shown in Figure 2B, the infectivity of 2OHβBCD-treated XMRV particles was not restored despite replenishing cholesterol. As previously described [9], partial cholesterol depletion of HIV-1 with low concentration (10 to 20 mM) of 2OHβBCD treatment reduced or blocked viral infection and this effect could be reversed by replenishing cholesterol by incubation of the treated particles with cholesterol/BCD complexes (Figure S1). Thus our observations for XMRV are in contrast to our previous findings for HIV and suggest that there are structural and/or functional differences between HIV-1 and XMRV with respect to virion-associated cholesterol.

### Analysis of 2OHpβCD-treated XMRV by Electron Microscopy

**Figure 3:**
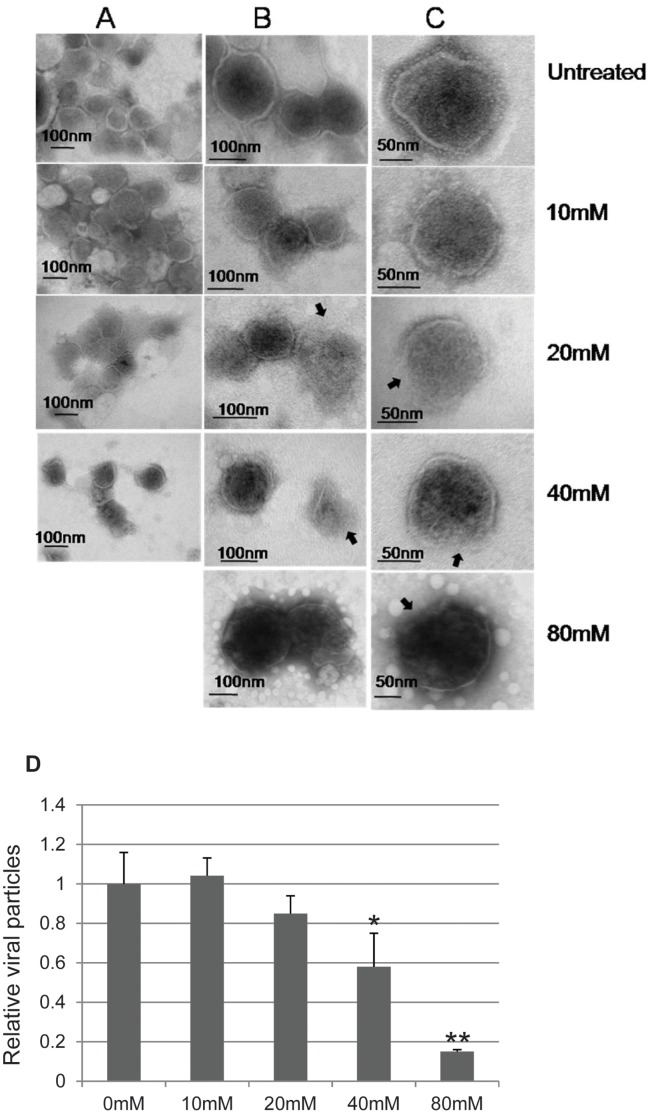
Electron microscopy analysis of 2OHpβCD-treated XMRV. XMRV samples, untreated or treated with 10, 20 and 80 mM 2OHpβCD, were examined by transmission electron microscopy. (**A**) Magnification: 6,000X–8,800X; (**B**) magnification: 8,800X–14,000X; (**C**) magnification: 14,000X–23,000X. (**D**) Quantitation of virions. Virions in the grid were quantified by counting 30 random fields for each concentration of 2OHpβCD-treated XMRV and the untreated control from three independent experiments. The relative number of viral particles is normalized to untreated control (arbitrarily set as 1). *P<0.05, **, *P*<0.001, compared to virion count from the untreated control.

The irreversible loss of viral infectivity of XMRV caused by 2OHpβCD suggests that cholesterol removal from XMRV results in a permanent change in virion structure that prevents infection. To test this possibility, we examined the morphology of XMRV particles after treatment with 10, 20 and 80 mM 2OHpβCD by transmission electron microscopy. Untreated virions were included as controls. At lower magnification, XMRV particles treated with 10 mM 2OHpβCD showed morphology very similar to that of control untreated virus (Figure 3 A and B, top two panels). There were differences in morphology between the 2OHpβCD-treated and untreated control groups when viewed at higher magnification. “Lolli-pop”-like Env spikes on the outer membrane were easily detected in the control untreated virus (Figure 3C, top panel). In contrast, very few if any of these structures were present in XMRV samples treated with 2OHpβCD at 10 mM or higher (Figure 3C). These results suggested that depletion of cholesterol from XMRV virions leads to loss of Env proteins from the viral membrane.

In previous studies, we demonstrated that treatment with higher concentrations of 2OHpβCD (≥40 mM) could lead to permeabilization of HIV-1 and leave large holes in the viral membrane [8]. We noted similar changes in 2OHpβCD-treated XMRV. The gaps or discontinuities in the viral membrane were seen in viral particles exposed to ≥20 mM 2OHpβCD (see arrows in Figure 3), whereas XMRV treated with 10 mM 2OHpβCD showed intact membranes as seen in untreated virus. The number of viral particles remaining on the grids was reduced after treatment with 2OHpβCD in a dose-dependent manner (Fig. 3D). At 80 mM 2OHpβCD, only a few virion-like structures could be found throughout the whole grid, and strikingly, these virions were swollen with a diameter in the range of 170–300 nm, 2–3 fold larger than the diameter of the untreated virions (Figure 3C, D). Taken together, these results show that treatment with higher concentrations of 2OHpβCD (≥20 mM) compromises XMRV membrane integrity and at very high concentrations disrupts the virions as previously reported for HIV and SIV.

### 2OHpβCD Treatment of XMRV Results in Sequential Loss of Envelope (gp70) and Capsid (p30) Proteins

**Figure 4:**
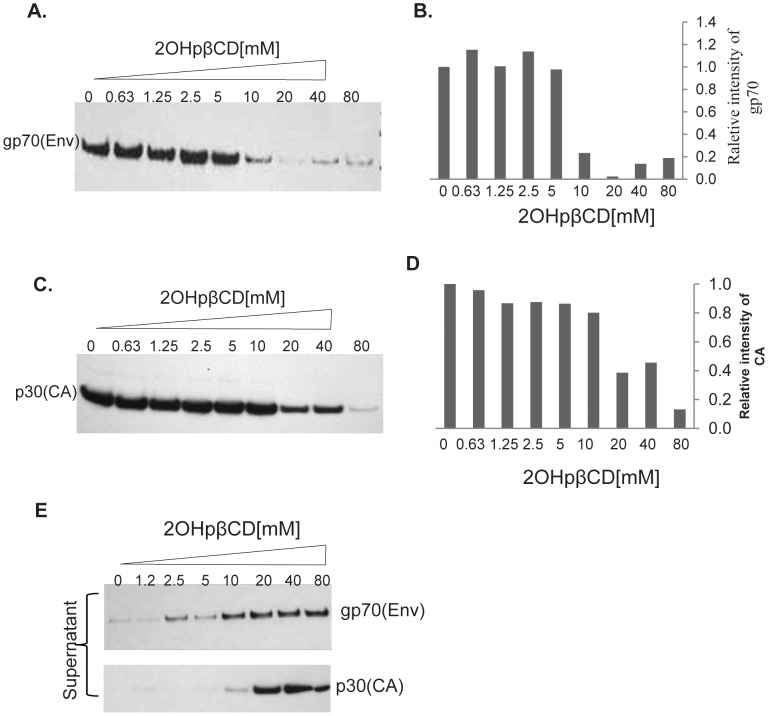
Cholesterol depletion leads to loss of viral Env and Gag in sequential pattern. (**A**) 2OHpβCD-treated XMRV was subjected to immunoblotting with a rat monoclonal antibody to SFFV Env. (**B**) The bar graphs reflect quantitative analysis of band intensities of (A); the results are expressed as relative band intensities, data are normalized to untreated virus (arbitrarily set as 1). (**C**) The same membrane was stripped and re-probed with goat antiserum to MLV p30 Gag. (**D**) The bar graphs reflect quantitative analysis of band intensities of (C), data are normalized to untreated virus (arbitrarily set as 1). (**E)** Env gp70 and and capsid p30 released into the supernatant after treatment of XMRV with 2OHpβCD was determined by immunoblotting of the post-treatment supernatants. Data are representative of five independent experiments.

The data shown above demonstrated that treatment of XMRV with 2OHpβCD could abolish infectivity and compromise the integrity of the viral membrane. To further address the effects of 2OHpβCD on the major structural proteins of XMRV, virions treated with increasing concentrations of 2OHpβCD were analyzed by immunoblotting with goat antiserum to MLV p30 Gag, and a rat monoclonal antibody to the spleen focus-forming virus (SFFV) Env protein. These antibodies show strong cross-reactivity with the equivalent XMRV proteins. As shown in Figure 4A, treatment of XMRV with 10 mM 2OHpβCD resulted in approximately 80–90% loss of the gp70 Env protein, compared to the untreated control. A higher dose of 2OHpβCD did not completely remove gp70 Env and a weak Env signal still remained even after treatment with 80 mM 2OHpβCD (Figure 4A). Quantitation of gp70 band intensities is shown in Figure 4B. The same blot was stripped and re-probed with anti-MLV p30 antibody. The amount of p30 protein was reduced about 50% and 90% by 2OHpβCD treatment at 20 mM and 80 mM, respectively, whereas treatment with 10 mM 2OHpβCD did not affect levels of the protein (Figure 4C). Quantitation of p30 band intensities is shown in Figure 4D.

Since exposure of XMRV to increasing concentrations of 2OHpβCD led to a progressive loss of virion–associated gp70 Env and Capsid (CA) p30, we determined whether Env and CA proteins were released into supernatant following virus treatment with 2OHpβCD. Env gp70 was detected at levels above baseline in cell supernatants following treatment with 2OHpβCD starting at 2.5 mM and the amount of gp70 detected significantly increased at 10 mM or higher (Figure 4E, top panel). This result correlated very well with the loss of Env gp70 from 2OHpβCD-treated virions where the highest loss occurred at concentrations of 10 mM or higher. The same blot was stripped and re-probed with anti-CA p30 antibody. Virion-associated capsid protein was more resistant to 2OHpβCD treatment compared with the envelope protein. Release of p30 protein was detected following treatment with 2OHpβCD starting at 10 mM and the signal was significantly increased at 20 mM or above (Figure 4E, bottom panel). Taken together, these findings demonstrate that depletion of cholesterol from XMRV results in loss of gp70. At higher concentrations of 2OHpβCD (≥20 mM), the capsid protein is lost as well, which is consistent with permeabilization of the virus. These results are in contrast to previous work on HIV-1 where depletion of cholesterol did not result in loss of gp120 or gp41 [8], [10]. We confirmed previous results where substantial loss of p24 is observed at concentrations of 2OHβBCD of 40 and 80 mM, whereas the signals for gp120 and gp41 remain unchanged at these concentrations (Figure S2).

### Analysis of other Structural Proteins and Viral RNA in 2OHpβCD-treated XMRV

**Figure 5:**
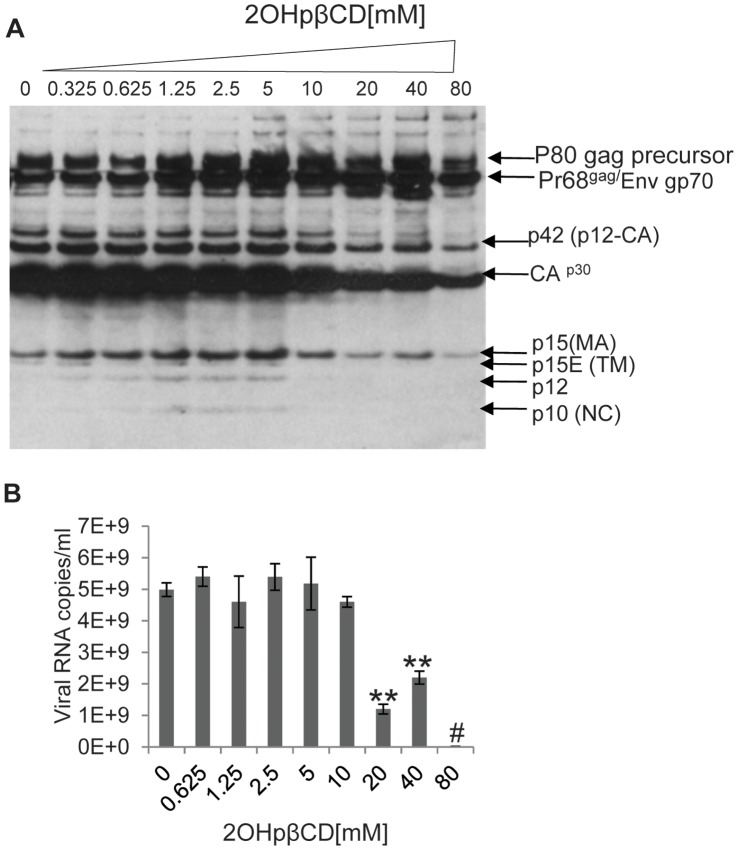
High concentrations of 2OHpβCD treatment results in loss of viral RNA and other structural proteins. (**A**) The same 2OHpβCD-treated XMRV as shown in Figure 4 was probed with goat antiserum to MLV. Data is representative of five independent experiments. (**B**) 2OHpβCD-treated XMRV was analyzed for particle-associated viral RNA by real-time RT-PCR. Error bars reflect standard derivation of three independent experiments. *, *P*<0.05, **, *P*<0.001, compared to the virions without 2OHpβCD treatment, #, below the threshold of detection.

Besides CA p30, XMRV has three additional mature core proteins derived from the Gag precursor protein: matrix (MA, p15), p12 and nucleocapsid (NC, p10). We examined the effects of 2OHpβCD treatment on these XMRV proteins. Due to the lack of an antibody specific to each of the proteins, we probed for them with goat polyclonal antiserum to Friend MLV. This polyclonal antiserum has been shown to detect all structural proteins of XMRV [27]. Similar to CA p30, the signal of virion-associated matrix protein (MA, p15) was reduced with 2OHpβCD treatment at 20 mM and was further reduced at 80 mM (Figure 5A). The signal for p12 and nucleocapsid (NC, p10) could be detected in the untreated control virus and in virus treated with 2OHpβCD at concentrations ranging from 0.325 mM to 5 mM. However, the signals for these two proteins were below the limit of detection in virions treated with 2OHpβCD at concentrations greater than 10 mM. A weak signal for the transmembrane subunit of envelope protein (TM, p15E, 14 kDa) was lost at concentrations of 2OHpβCD of 5 mM or higher (Figure 5A).

We next determined whether viral RNA was retained in XMRV following 2OHpβCD treatment. After exposure to different concentrations of 2OHβCD, virions were lysed and subjected to qRT-PCR analysis with primers for the gag leader region. There was a substantial reduction in the amount of RNA at 2OHpβCD concentrations at 20 mM or above. The RNA in virus treated with 80 mM 2OHpβCD was close to the threshold of detection (Figure 5B). Overall these data are consistent with other findings indicating that depletion of virion-associated cholesterol disrupts membrane integrity resulting in loss of internal viral components.

### Cellular Cholesterol Depletion Impairs the Release of XMRV from Infected Cells but does not Change Infectivity of Progeny Virus

**Figure 6:**
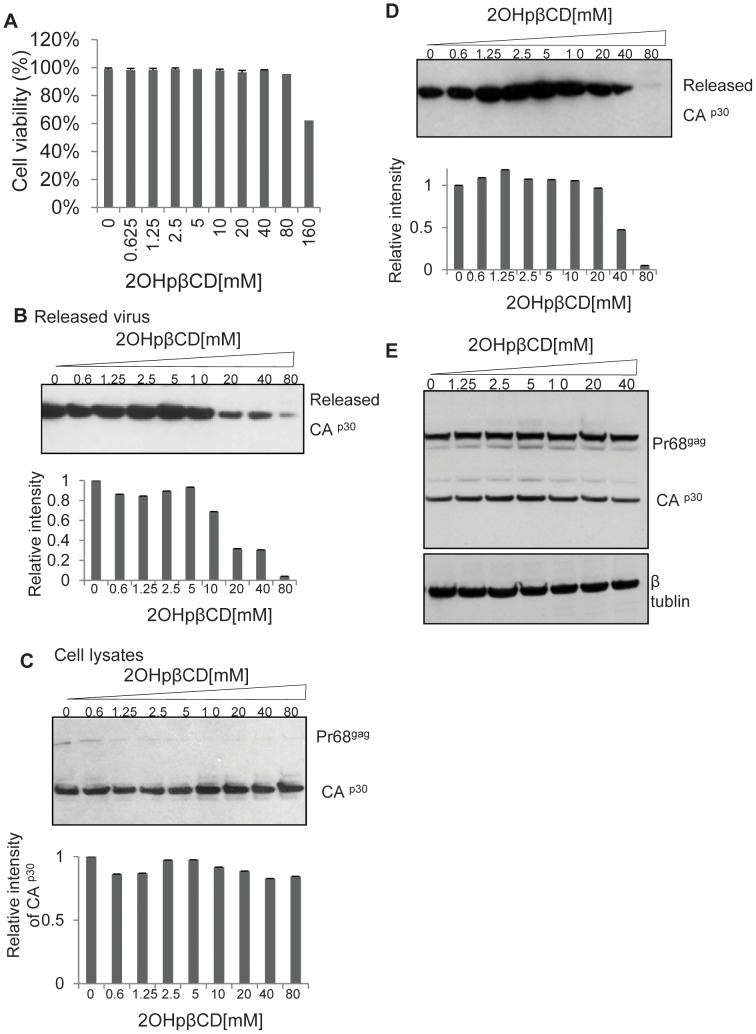
2OHpβCD treatment impairs XMRV release from LNCaP cells. LNCaP cells chronically infected with XMRV were treated with increasing concentrations of 2OHpβCD for 1 hr at 37°C, after which cholesterol free medium was replaced and the released virus was collected 5 hrs later. (**A**) The cell viability with different concentration of 2OHpβCD treatment was determined by 7AAD staining followed by flow cytometry, the quantified data are normalized to untreated cells, (arbitrarily set as 100%). (**B**) The released virus and (**C**) cell lysates were subject to the Western blot with anti-p30 Gag antibody. (**D**) After treatment of with 2OHpβCD, the LNCaP-XMRV cells were washed and resuspended in RPMI containing 1 mM 2OHpβCD pre-complexed with cholesterol (48 µg/ml) and incubated for 2 hrs, after which cholesterol free medium was replaced. After a 5 hrs incubation the released virus was collected and subjected to the Western blot analysis with anti-p30 Gag antibody. Data are representative of five independent experiments. The band intensities of (B), (C) and (D) were shown in the bottom of each blot (quantified and normalized to untreated infected cells, arbitrarily set as 1). **E**, The virus released from 2OHpβCD treated LNCaP-XMRV was normalized by RT and used to infect naïve LNCaP cells; the viral infectivity was determined by viral protein expression in the infected cells with goat antiserum to MLV p30 Gag.

Lipid rafts and cholesterol has been shown to play critical roles in HIV-1 assembly and release. To determine the role of cholesterol in XMRV release, we treated LNCaP cells chronically infected with XMRV with increasing concentrations of 2OHpβCD or medium as a control for 1 hr at 37°C. Cell viability was not affected by 2OHpβCD treatment at concentrations of 40 mM or below as compared to control. Viability was slightly reduced at 80 mM and significant cell toxicity was observed at 160 mM (Figure 6A). Treated cells were then washed, and cultured for 5 hrs in serum-free, cholesterol-free medium to allow production of XMRV. The virus-containing supernatants and cell lysates were subjected to the western blot analysis. As shown in Figure 6B, the signal of CA p30 released into the culture medium was greatly reduced by treatment with 2OHpβCD at 20 mM or above. In contrast to the viral associated CA p30, the level of cell-associated CA p30 was not affected by 2OHpβCD treatment (Figure 6 C).

We next determined whether the effect of 2OHpβCD treatment on virus release could be reversed by replenishing depleted cellular cholesterol. We treated XMRV-infected LNCaP cells with 2OHpβCD as described above, washed the cells and then incubated them in medium alone or in medium containing 48 µg/ml of cholesterol in a pre-formed complex with 1 mM 2OHβBCD for 2 hrs at 37°C. The cells were then washed and allowed to produce virus for 5 hrs as described above. The virions released into the supernatant were subjected to western blot analysis. As shown in Figure 6D, the release of virions from the cells that had been treated with 20 or 40 mM 2OHpβCD was almost completely restored (20 mM) or partially restored (40 mM) by adding back cholesterol. Virus release could not be restored by exogenous cholesterol in cells treated with 80 mM 2OHpβCD.

Previous work on HIV-1 suggested that cholesterol depletion of the infected cells rendered the progeny virions non-infectious [9], [12]. We examined the infectivity of XMRV released from infected cells that had been treated with 2OHpβCD. The virus preparations from treated cells were normalized for reverse transcriptase activity and tested for infectivity on LNCaP cells. Virus infection was determined by measuring the cellular expression of viral proteins by immunoblotting and by assaying reverse transcriptase activity in cell culture supernatants. Our data show that the infectivity of progeny virus from the 2OHpβCD-treated cells was very similar to that of progeny virus from control untreated cells (Figure 6 E). So while cholesterol depletion greatly suppressed virus release from cells, the released virions retained their infectivity.

## Discussion

Pathogenic retroviruses are etiologic agents of human diseases such as leukemia and AIDS [28]. Extensive previous studies of HIV-1 by our laboratory and by others have shed light on common biological properties of retroviruses, such as the importance of lipid rafts in early and late viral events and the essential role of cholesterol in retrovirus replication. In this study we sought to determine whether the gammaretrovirus XMRV shared these features in common with HIV-1 and other retroviruses. We have examined the effect of cholesterol depletion by 2OHpβCD on the structure and infectivity of XMRV and we examined the role of cell-associated cholesterol on XMRV release.

Measuring the residual cholesterol in 2OHpβCD-treated XMRV indicated that cholesterol loss happened at concentrations of 10 mM or higher and this coincided with loss of viral infectivity that could not be restored by replenishing cholesterol. This result was different from previous studies on HIV-1 in which partial depletion of cholesterol from HIV-1 with 2OHpβCD abrogates infectivity which could be restored by replenishing viral cholesterol [8], [9]. Differences were further reflected by analyzing treated virus by EM and immunoblotting. After 10 mM 2OHpβCD treatment, which resulted in depletion of 30–40% of cholesterol associated with XMRV, virions remained intact and core proteins were retained but viral envelope glycoprotein (gp70) was lost. These results were somewhat surprising since in previous work on HIV-1 cholesterol could be substantially removed (60–80%) from virions without disrupting their membrane integrity and Env protein was retained [8], [9], [10]. Like HIV-1, MLV Env protein is palmitoylated resulting in lipid raft localization [17], [29], [30], [31]. The loss of XMRV envelope protein after cholesterol depletion and disruption of lipid rafts indicates that XMRV Env may not have raft-independent interactions with other viral proteins as are found in other retroviruses. Consistent with this idea, a recent study found that MLV Gag has a much weaker propensity for inter-protein interactions than HIV-1 Gag [32], [33]. Weaker interactions on the part of XMRV Gag could possibly play a part in the apparent weaker association of gp70 Env with XMRV particles. Our observations are consistent with a previous study on E-MLV in which Pickl et al reported that removal of cholesterol from the virus with methyl-β-cyclodextrin abolished viral infectivity in a dose dependent manner. Interestingly, they found that heterologous Env expressed on pseudotyped MLV is more sensitive to loss after cholesterol depletion; they proposed a weaker association heterologous Env proteins with MLV particles [34]. XMRV appears to have been generated by recombination between two endogenous viruses [26], [35]. Whether this recombination event resulted in less stable association between Env and core viral proteins is unknown. The definitive delineation of the exact role of cholesterol and lipid rafts in Env protein association with viral capsids and how this varies in different viruses will require further study.

In the present study the infectivity of XMRV was below threshold levels of detection after treatment with 2OHpβCD at concentrations greater than 10 mM. The loss of viral infectivity is likely due to loss of Env protein but we cannot exclude the possibility that other effects such as loss of fusion capacity also contributed [9]. We found that XMRV viral membrane integrity was compromised and permeabilization occurred when virions were treated with 2OHpβCD concentrations of ≥20 mM. Permeabilization correlated with complete abolition of infectivity, the removal 80–90% cholesterol from virions and the loss of internal viral proteins. In previous studies on HIV-1 we found that extensive cholesterol depletion from virus caused dissociation of virion-assoicated raft microdomains and the generation of non-infectious permeabilized virions characterized by the loss of mature viral core proteins (capsid and matrix) without loss of envelope glycoproteins [8], [9], [10]. The data from this current study are entirely consistent with and support these earlier findings for HIV-1 except the loss of Env protein as discussed above. We showed that infectivity of XMRV could not be restored by exogenous cholesterol and this was also in contrast to earlier data for HIV-1. These two observations are probably related as cholesterol alone would not overcome the need for a viral attachment/fusion protein even under conditions when viral RNA and core proteins are retained.

It is now clear that a number of viruses utilize lipid rafts as sites of assembly and release from infected cells. Several lines of evidence support a role for lipid rafts in MLV release: 1) Lipid profile analysis indicated that E-MLV and HIV-1 are highly enriched in lipids found in rafts [15]; 2) The association of Env with lipid rafts has been reported for E-MLV and A-MLV [16], [17], [18], [29]; 3) Depletion of cell-associated cholesterol with methyl-β-cyclodextrin impairs E-MLV release [36]. Here our results confirm and extend the role of lipid rafts in retrovirus biology to XMRV. Cholesterol depletion from the membrane of XMRV-infected cells with 2OHpβCD reduced virus release substantially. Unlike the effect of cholesterol depletion on XMRV particles, the effect of cholesterol depletion on XMRV infected cells could be reversed by exogenous cholesterol consistent with our previous finding for HIV-1. Interestingly, while we observed release of fewer XMRV particles from 2OHpβCD-treated cells, the particles were equally infectious to those released by control cells. This too was in contrast to previous work on HIV-1. Taken together these results suggest that while XMRV shares some features in common with HIV-1 relative to cholesterol and lipid rafts, there appear to be some important differences as well. Dissecting these differences may provide new insights into the involvement of cholesterol and rafts in retrovirus biology.

## Materials and Methods

### Cells and Reagents

The LNCaP cell line was purchased from American Type Culture Collection (Rockville, MD). LNCaP cells chronically infected with XMRV were obtained from Dr. Waldemar Popik, Meharry Medical College, Nashville, Tennessee. Cells were maintained in cRPMI [RPMI 1640 (Gibco-BRL/Life Technologies, Gaithersburg, MD) supplemented with L-glutamine, 10 mM HEPES (pH 7.2), and 10% fetal bovine serum (FBS) (Hyclone, Logan, Utah)]. 2OHpβCD was obtained from Cyclodextrin Technologies Development (CTD, Gainesville, FL).

### 2OHpβCD Treatment of Virus and Cells

XMRV was purified from culture supernatants of infected LNCaP cells by ultracentrifugation for 1 hr at 100,000×g, 4°C. Purified XMRV was resuspended in serum-free RPMI medium and a constant amount of virus was then incubated with 1 ml of increasing concentrations of 2OHpβCD in serum-free medium for 1 hr at 37°C. As a control, virus was treated with 1 ml of RPMI medium without 2OHpβCD. The virus was recovered by ultracentrifugation and resuspended in the original volume. The treated virus was then aliquoted and stored at −80°C. To determine whether 2OHpβCD affects viral production, LNCaP cells chronically infected with XMRV were washed with serum-free RPMI medium and treated with increasing concentrations of 2OHpβCD in serum-free medium for 1 hr at 37°C. The cells were then washed twice with PBS and incubated for 5 hrs at 37°C in cRPMI. The virus-containing supernatants were collected and filtered through a 0.45 µm syringe filter. Cell viability was determined by staining with 7-aminoactinomycin D (7-AAD) followed by flow cytometry analysis to identify positive (dead) cells.

To determine whether the depletion of cholesterol is responsible for the observed inhibition of XMRV infection or viral production, 2OHpβCD-treated virus or cells were incubated with cholesterol (48 µg/ml as a complex with 1 mM 2OHpβCD) for 1 to 2 hrs at 37°C to replenish cholesterol and then tested for XMRV infection or viral production [8], [9].

### Measurement of Cholesterol

Cellular and viral cholesterol content was measured with a cholesterol oxidase-based assay (Amplex Red, Molecular Probes). Cholesterol content was normalized to total protein as previously described [8], [9].

### Infectivity Assays

2OHpβCD-treated virions or virus in supernatants from 2OHpβCD-treated XMRV-infected LNCaP cells were quantified by total protein (BCA assay, Pierce) or by reverse transcriptase activity (Roche colorimetric reverse transcriptase assay). The virus was then used to infect LNCaP cells in cholesterol-free medium and 6–8 hrs post-infection the input virus was removed by washing the cells three times with PBS. Complete medium was then added and 48 hrs post-infection, Western blot and real time PCR were performed to assess cellular XMRV viral protein and viral RNA in supernatants or cellular pro-viral DNA, respectively, as a measure of productive infection.

### Western Blot Analysis

Proteins were separated on SDS-10% polyacrylamide gels and analyzed as described [37]. Antibodies for Western blot were obtained from the following sources: Rat monoclonal antibody against SFFV Env was kindly provided by Dr. Sandra K. Ruscetti (NIH, Bethesda, MD). Goat antiserum to XMLV or to MLV p30 Gag, and mouse hybridoma cell line secreting Mab against MLV p30 Gag were obtained from American Type Culture Collection (Rockville, MD). Densitometry was performed on scanned images of immunoblot films using the ChemiDoc™ XRS+ System or on the blots using ChemiDoc™ MP image system (Bio-Rad). Band intensities were analyzed with Quantify One or Image Lab software.

### Quantitation of Viral RNA and Proviral DNA

To determine whether XMRV RNA was retained in the virions after 2OHpβCD treatment and quantify viral production from infected cells, the viral RNA was isolated from purified virus or virus-containing supernatants using QIAamp Viral RNA mini Kit (Qiagen) and subjected to reverse transcription with SuperScript III cDNA synthesis kit (Invitrogen). To analyze the proviral DNA from infected cells, total cellular DNA was isolated from cells using DNAeasy DNA isolation kits (Qiagen). Quantified Real-time PCR (qPCR) was performed using iQsybr green supermix (Bio-Rad) (primers: 5′-AACCGTATGGCAGATCAAGC-3′ and 5′-TTTGCCTTGTAGGACCCAAT-3′). VP62 plasmid (NIH AIDS Research and Reference Reagent Program) was used as template to generate standard curves.

### Electron Microscopy

Solutions of 2OHpβCD-treated and untreated viruses were dropped onto a sheet of Parafilm, and Formvar-carbon coated grids were floated for 1 min at room temperature to adsorb viral particles. The grids were then washed in PBS, fixed with 2% paraformaldehyde and washed with PBS. After negative staining with 1% phosphotungstic acid for 30 sec, the samples were analyzed using a JEOL transmission electron microscope at 80 kV.

### Statistical Analysis

All experiments were performed three to five times, and representative experiments are shown. Statistical significance was determined using the Student *t* test.

## Supporting Information

Figure S1 **Infectivity of low concentration of 2OHpβCD-treated HIV-1 is restored by replenishing cholesterol.** Concentrated HIV-1 particles were treated with 2OHpβCD then exposed to RPMI containing cholesterol (48 ug/ml as a complex with 1 mM 2OHpβCD) (BCD/Cholesterol) for 1 hr at 37°C, the treated virus exposed to medium alone served as control (BCD/Medium). (**A**) The cholesterol in virions was quantified by Amplex Red assay, the relative cholesterol is normalized to untreated control (0 mM, arbitrarily set as 100%). The data shown represent the mean ± standard deviation from three independent experiments. *, *P*<0.05; **, *P*<0.001 (virions treated with BCD/medium versus virions treated with BCD/Cholesterol. (**B**) The virus preps were normalized by p24 ELISA and the infectivity was determined on TZM-bl cells by measuring luciferase activity. Error bars reflect standard derivation of three independent experiments.
(TIF)

Figure S2 **Cholesterol depletion does not release HIV-1 Envelope proteins from virions.** Concentrated HIV-1 particles were treated with 2OHpβCD as described before. The treated samples were then subjected to Western blot analysis for HIV-1 proteins (Gag p55 and p24, gp41 and gp120) as indicated. Data are representative of three independent experiments
(TIF)
