# Supplementary figures and images for: Cholesterol Depletion Inactivates XMRV and Leads to Viral Envelope Protein Release from Virions: Evidence for Role of Cholesterol in XMRV Infection
Source: PLoS One. 2012 Oct 26;7(10):e48013. doi: 10.1371/journal.pone.0048013 (PMC3482229; doi:10.1371/journal.pone.0048013)

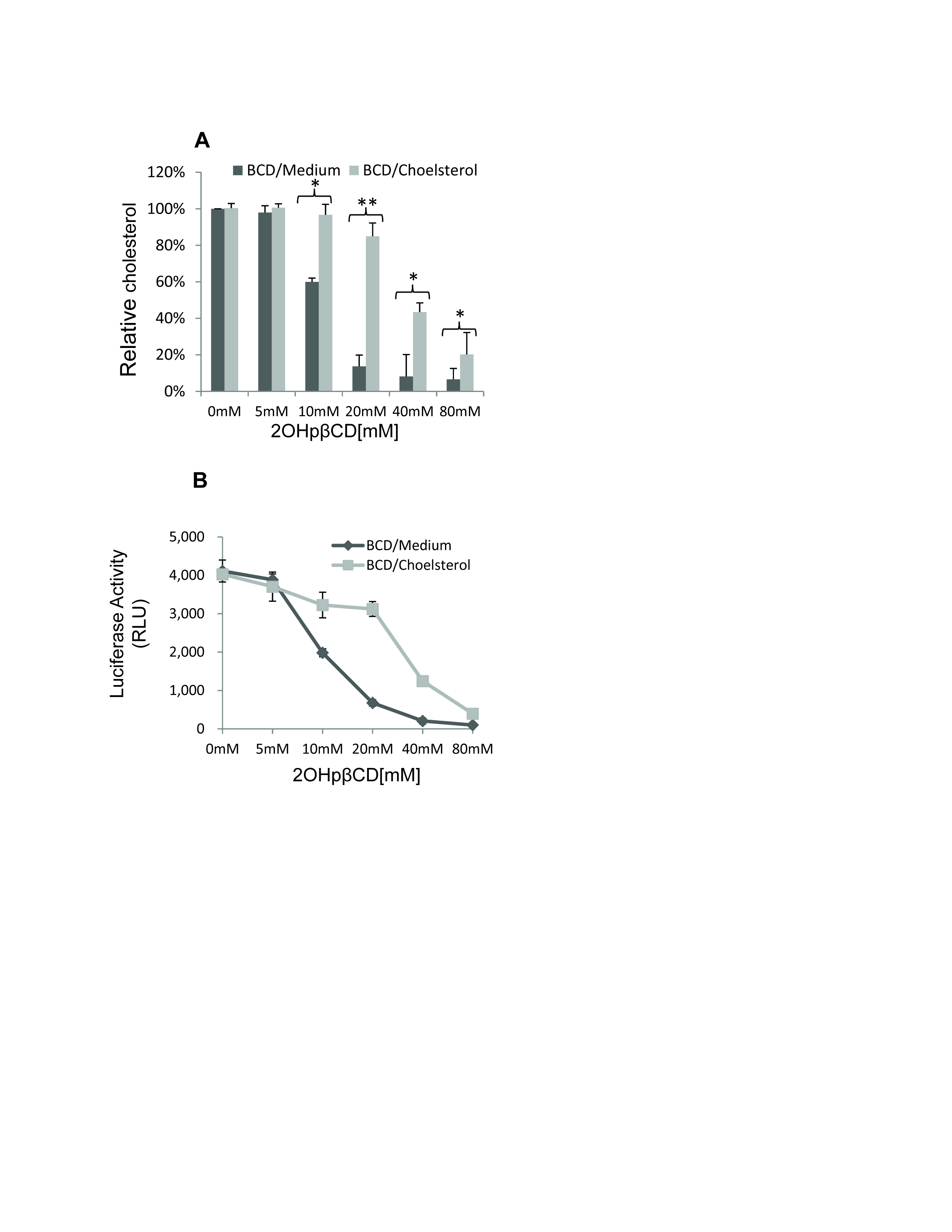

Supplement: Figure S1 — Infectivity of low concentration of 2OHpβCD-treated HIV-1 is restored by replenishing cholesterol. Concentrated HIV-1 particles were treated with 2OHpβCD then exposed to RPMI containing cholesterol (48 ug/ml as a complex with 1 mM 2OHpβCD) (BCD/Cholesterol) for 1 hr at 37°C, the treated virus exposed to medium alone served as control (BCD/Medium). (A) The cholesterol in virions was quantified by Amplex Red assay, the relative cholesterol is normalized to untreated control (0 mM, arbitrarily set as 100%). The data shown represent the mean ± standard deviation from three independent experiments. *, P<0.05; **, P<0.001 (virions treated with BCD/medium versus virions treated with BCD/Cholesterol. (B) The virus preps were normalized by p24 ELISA and the infectivity was determined on TZM-bl cells by measuring luciferase activity. Error bars reflect standard derivation of three independent experiments. (TIF) [file pone.0048013.s001.tif]

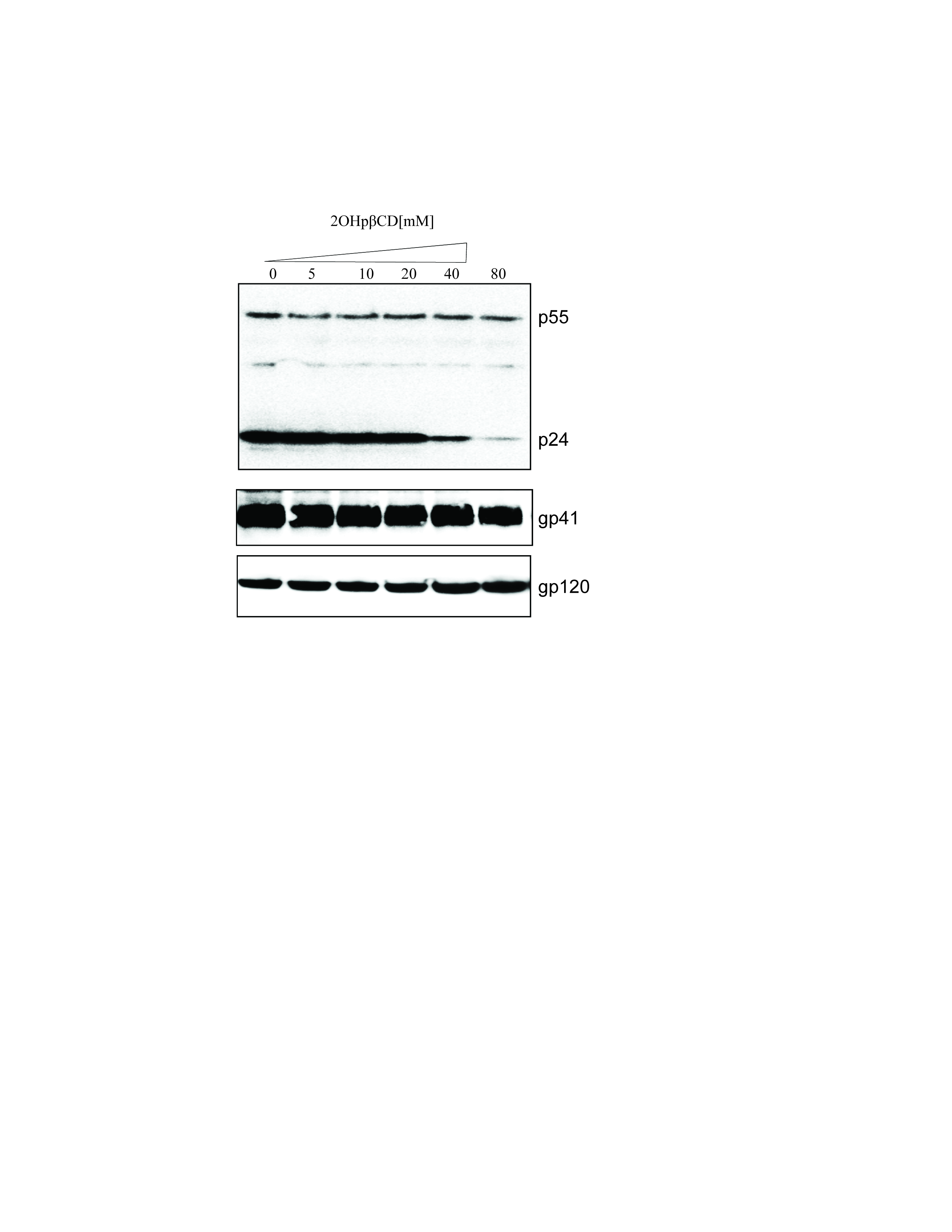

Supplement: Figure S2 — Cholesterol depletion does not release HIV-1 Envelope proteins from virions. Concentrated HIV-1 particles were treated with 2OHpβCD as described before. The treated samples were then subjected to Western blot analysis for HIV-1 proteins (Gag p55 and p24, gp41 and gp120) as indicated. Data are representative of three independent experiments (TIF) [file pone.0048013.s002.tif]
